# Conservative management of esophageal perforation following obesity surgery

**DOI:** 10.1590/S1516-31802006000600008

**Published:** 2006-11-01

**Authors:** José Celso Ardengh, Carlos Eduardo Domene, Loana Heuko Valiati, Alexander Charles Morrell

**Keywords:** Obesity, Surgery, Esophageal, perforation, Bariatrics, Esophagus, Obesidade, Cirurgia, Perfuração esofágica, Bariatria, Esôfago

## Abstract

**CONTEXT:**

Laparoscopic adjustable silicone gastric banding (LASGB) is one of the several surgical techniques for treating patients with morbid obesity. Erosion and perforation in the gastric chamber caused by LASGB are rare complications that have already been described. There have not yet been any reports of perforation of the middle esophagus during this procedure.

**CASE REPORT:**

The authors describe the case of a patient who presented the complication of very extensive perforation of the middle third of the esophagus following LASGB. This was successfully managed using conservative treatment.

## RESUMO

**CONTEXTO:**

Banda gástrica laparoscópica ajustável de silicone (LASGB) é uma das várias técnicas cirúrgicas para o tratamento de pacientes com obesidade mórbida. A erosão e a perfuração para o interior da câmara gástrica causados pela LASGB são complicações raras já descritas. Não se encontram relatos de perfuração do esôfago médio durante esse procedimento.

**RELATO DE CASO:**

Descrevemos o caso de uma paciente que apresentou como complicação, uma perfuração extensa do esôfago médio após LASGB, submetida a tratamento conservador com sucesso absoluto.

## INTRODUCTION

Laparoscopic adjustable silicone gastric banding (LASGB) is one of the several bariatric surgical techniques for patients with morbid obesity. Because it is less invasive and presents lower rates of intraoperative and postoperative systemic complications, it has been widely indicated.^1,2^ Erosion and perforation in the gastric chamber caused by LASGB are rare complications that have already been described.^1,2^

There have not yet been any reports of perforation in the middle esophagus caused by this procedure. Searches in Medline (Medical Literature Analysis and Retrieval System Online) and Lilacs (Literatura Latino-Americana e do Caribe em Ciências da Saúde) did not yield any such reports. This fact, together with the unusual evolution of the present case (without requiring surgery), vests great scientific interest in the publication of this case.

Most cases of esophageal perforation occur as a result of diagnostic endoscopy, therapeutic endoscopy, foreign body ingestion^3^ and inadvertent intubation with an orotracheal tube in the esophagus.^4^ Bleeding, mediastinitis and pyothorax may develop after perforation occurs.^3,4^ The present authors report on the previously unpublished occurrence of a case of esophageal perforation in the middle third of the esophagus that was managed with conservative treatment.

## CASE REPORT

E.S.M., a 47-year-old female, was admitted 56 hours after morbid obesity treatment using LASGB. Immediately after the obesity surgery, she started to present severe retrosternal pain, which became worse after deglutition and deep inspiration. Physical examination showed that her temperature was 35.4° C. There was no evidence of bleeding. She had minor cervical and thoracic subcutaneous emphysema. Pulmonary auscultation detected breathing sounds bilaterally, with signs of pleural effusion on the left side. Abdominal examination showed five incisions that had been sealed using continuous stitches.

The laboratory tests upon admission showed a blood sedimentation rate of 80 mm/h, and the hemogram presented a leukocyte count of 17,700/μl, with 78% neutrophils.

**Figure 1:**
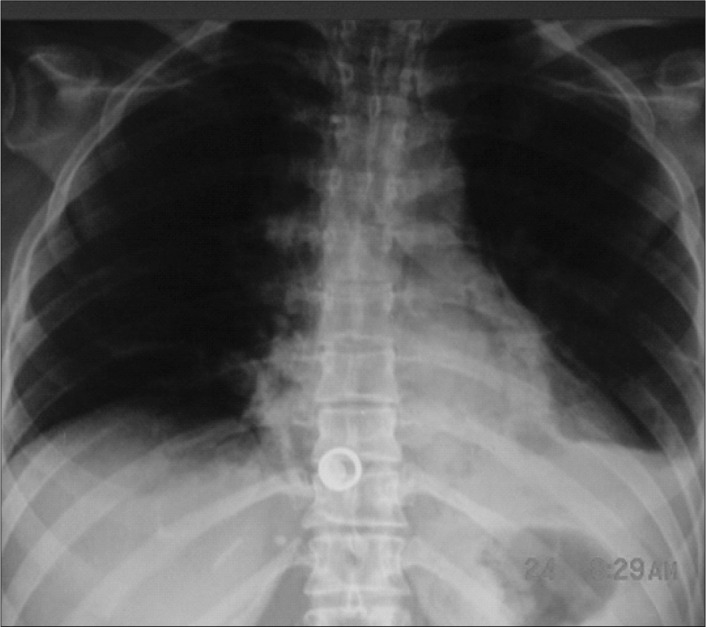
Chest x-ray showing left pleural effusion and pneumomediastinum in a woman operated for morbid obesity.

The initial x-ray, and also tomography with contrast medium, showed the presence of pneumomediastinum and extravasation of contrast from the esophagus. No presence of air below the diaphragmatic cupola was observed (Figure 1).

**Figure 2:**
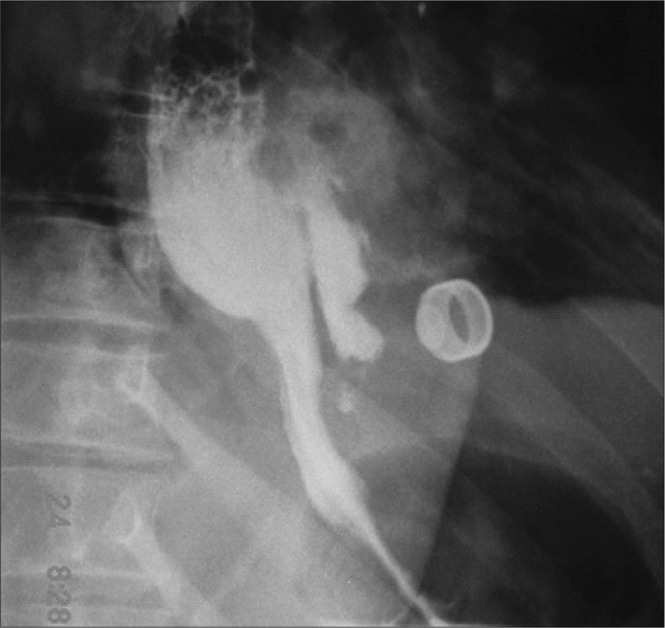
Esophagogram upon admission of a woman operated for morbid obesity, with suspicion of esophagus perforation. Note the extravasation of contrast, in the middle esophagus, as far as a blind end in the mediastinum.

To confirm the diagnosis of esophageal perforation, we obtained an esophagogram upon admission and performed a control thirteen days after treatment (Figure 2).

**Figure 3:**
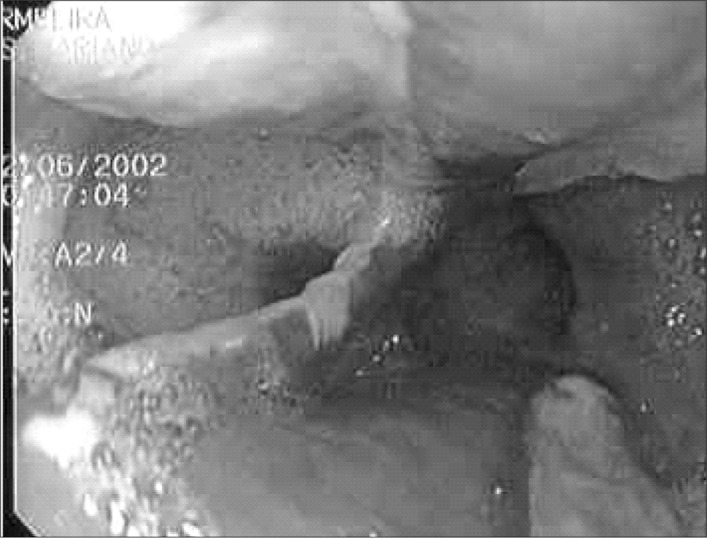
Two linear and longitudinal ulcers covered with fibrin, located in opposite walls, in the esophagus of a woman operated for morbid obesity, as showed by endoscopy.

We also performed upper digestive endoscopy to evaluate the extent and depth of the perforation. This examination showed the presence of a deep perforation located on the left lateral wall, with a length of 5.0 cm, starting at a distance of 30 cm from the anterior incisors (Figure 3).

These findings suggested that perforation had occurred following dilation of the balloon that was used for calibration during LASGB placement. Since there were no signs of local abscess, and because the patient was in a good clinical condition and the size of the perforation allowed good local drainage without any fluid collection, we chose to place the patient on prolonged fasting and introduce a nasoenteral tube into the duodenum. This conservative treatment consisted of hypernutrition by nasoenteral tube, proton pump inhibition (omeprazole 40 mg/day, intravenously) and antibiotics (ceftriaxone 1 g/day and metronidazole 400 mg/day, both intravenously) for fourteen days, respiratory physiotherapy and inhalation of 9% physiological serum via bronchodilator. We did not use either a nasogastric or a nasoesophageal tube.

**Figure 4:**
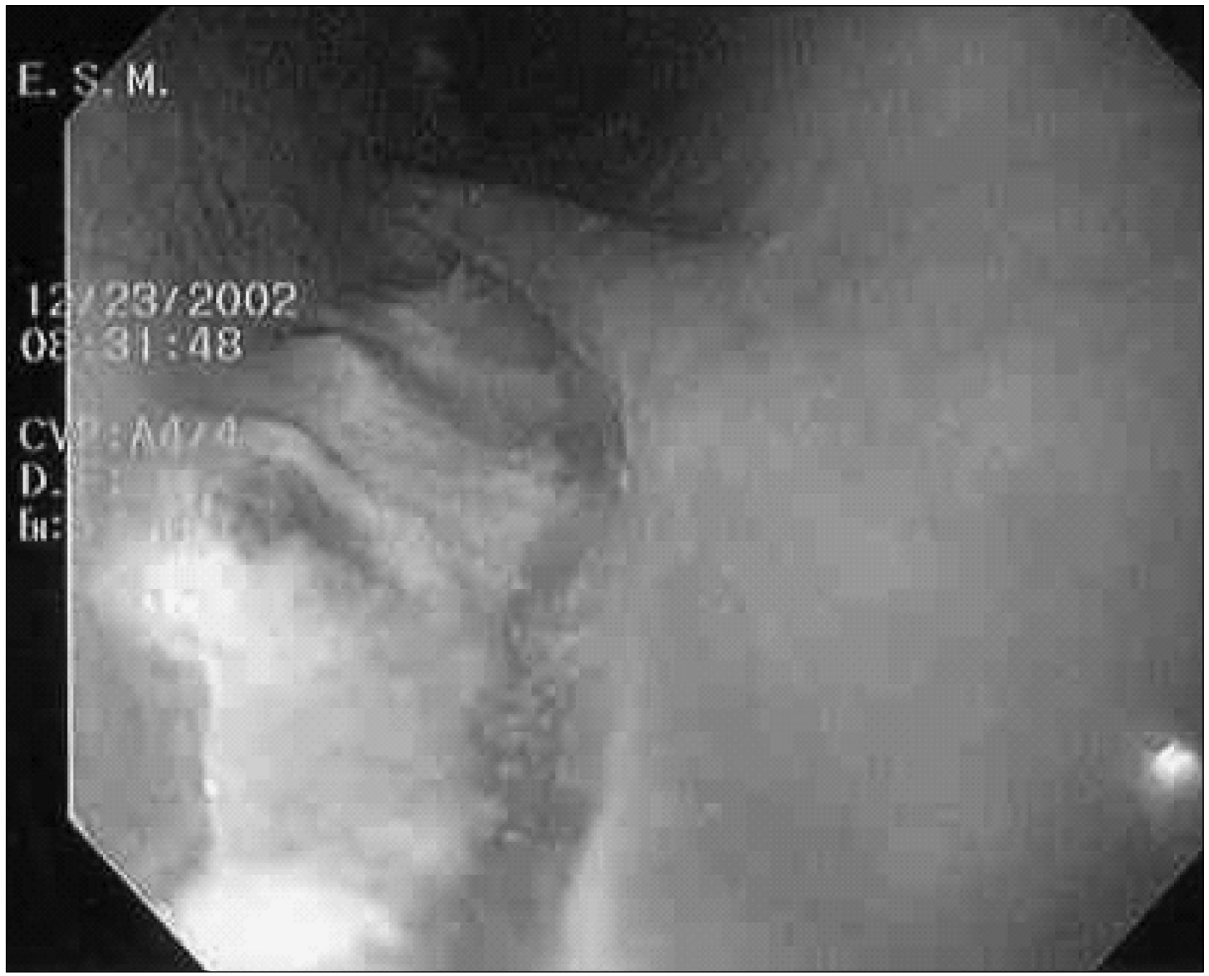
Endoscopic view of an ulcer of 4.5 cm in length in the esophagus, covered with granulation tissue, fifteen days after starting conservative treatment (initial length was 5.0 cm).

By three days after admission, the patient's symptoms had disappeared. She did not present fever and her vital signs were stable. All the laboratory tests gave normal results. The absolute leukocyte count was 8,900/µl, with 55.2% neutrophils. Endoscopy performed after 15 days showed an ulcer of 4.5 cm in length, with a clean base and granulation tissue (Figure 4). The patient was then allowed to start taking a liquid diet, which had good acceptance. After 38 days, we performed endoscopy again, which showed that the wound had completely healed. The patient continues to have no symptoms or sequelae from the perforation.

## DISCUSSION

Many reports have demonstrated that the laparoscopic approach is safe and presents good results. However, it needs to be emphasized that there is an intimate relationship between the surgeon's experience and the obtainment of good results and low complication rates.^5^

Fielding et al.^6^ reported their results from 335 patients who underwent LASGB. Twenty of them (6%) needed reoperation because of gastric prolapse of the band. Most of these bands were removed because of reflux and food intolerance. These authors report only one case of perforation, which was in the gastric fundus, when the band was removed.

O'Brien et al.^7^ studied 302 patients with gastric bands prospectively. The incidence of early complications was 4%, including two cases of gastric perforation: one in a case of open surgery and the other in a case of gastric reservoir infection. Gastric mucosa prolapse inside the band occurred in 9% and was the main late complication. These authors concluded that LASGB provided short hospital stay, low complication rate and effective weight loss.

Another frequently described complication is erosion of the gastric chamber caused by the band, which occurs in 0.31%, six to eight months following the procedure. Most of such patients heal after band removal.^1^ Other complications described have been: abscess formation at the port location, gastric fistula and subphrenic collection of inflammatory liquid.^1^

Esophageal perforation is relatively rare. It may occur as a result of diagnostic endoscopy, therapeutic endoscopy or esophageal instrumentation.^3^ Until the present report, there had not been any report in the literature regarding this type of complication during LASGB placement, which made publication of this case an attractive proposition. Soto et al.^8^ presented the case of a patient with lower esophageal perforation that apparently resulted from orogastric calibration tube passage during laparoscopic placement of a gastric band.

Perforation of the middle third of the esophagus, as in our patient, may lead to the development of mediastinitis, pericarditis, or pleural empyema due to left pleural effusion.^3,4^ In our patient, the perforation mechanism was the same as described in cases of pneumatic balloon dilation for achalasia, when the dilation balloon is insufflated in an undiseased organ, causing internal and radial forces that break the wall in a fragile portion and cause ulcers in the other walls. Infection usually occurs after 24 to 48 hours.^4^

Although most cases of esophageal perforation are treated surgically, the mortality rate is around 22%.^4^ The fundamental prognostic factors in the evolution of esophageal perforation are the length of time between the event and its diagnosis, the patient's clinical condition and the perforation characteristics (size, depth and presence of local inflammation or sepsis). Patients who receive treatment within 24 hours of the onset of symptoms have a higher survival rate (up to 92%). On the other hand, patients who receive treatment only after 24 hours have elapsed have a mortality rate ranging from 40 to 50%. Many complications may occur as a result of late treatment, i.e. more than 24 hours after the triggering event.^5^

Recently, clinical treatment through the use of antibiotic therapy and prolonged parenteral nutrition has been reported in a small number of cases of esophageal perforation.^3^ In our case, certain factors were fundamental for obtaining good evolution: antibiotic administration during the surgery, the patient's good clinical and nutritional condition, and the size and depth of the lesion, which enabled extensive drainage without the occurrence of mediastinal fluid collection.

It is important to note that early diagnosis and intervention may modify the clinical evolution of cases of esophageal perforation, thereby reducing the morbidity and mortality rates. Most cases have a poor prognosis, and immediate surgical intervention is necessary.

General physicians and surgeons should bear in mind that conservative clinical treatment for esophageal perforation can be implemented in cases without signs or symptoms of infection or sepsis, when there is simple perforation caused by probes or medical i nstruments or foreign body ingestion.^3^

In some cases of extensive deep perforation, as in our case, early diagnosis can make it possible for such patients to improve with conservative clinical treatment, dependent solely on whether they are in a good general condition.
